# Genetic and environmental influences on the germination of basidiospores in the *Cryptococcus neoformans* species complex

**DOI:** 10.1038/srep33828

**Published:** 2016-09-20

**Authors:** Adrian Forsythe, Aaron Vogan, Jianping Xu

**Affiliations:** 1 Department of Biology, McMaster University, 1280 Main St West, Hamilton, Ontario, L8S 4K1, Canada

## Abstract

In basidiomycetous fungi, the viability of basidiospores is an important component of sexual fitness. However, relatively little is known about the genetic and environmental factors influencing basidiospore germination. In this study, we used human opportunistic yeast pathogens, *Cryptococcus neoformans* and *Cryptococcus deneoformans*, as models to investigate the potential effects of selected genetic and environmental factors on basidiospore germination. A total of five strains with known genome structure were used to construct six crosses, three of which were between strains within the same species, while the remaining three were hybrid crosses between *C. neoformans* and *C. deneoformans*. Offspring from these crosses were incubated on two media (a nutrient-limiting and a nutrient-rich) and three temperatures (23 °C, 30 °C, and 37 °C). In general, spores from intra-specific crosses had greater germination rates than those from inter-specific crosses. Of the two environmental factors, temperature showed a greater influence than nutrient medium, with the 37 °C environment yielding lower germination rates than at 23 °C and 30 °C environments in most crosses. Furthermore, there were notable interaction effects between environmental factors and parental strains or strain pairs on basidiospore germination. We discuss the implications of these results on pathogenesis and speciation in this human fungal pathogen.

In sexual eukaryotes, the viability of gametes is a key component of their reproductive fitness and low gamete viability is frequently used as an indicator of post-zygotic reproductive isolation between parental populations. In contrast to pre-zygotic reproductive isolations that are commonly mediated by physical, temporal, and/or mechanical barriers, post-zygotic reproductive isolations are caused by genetic factors^1^. At present, most studies on the mechanisms of post-zygotic reproductive isolation have involved model systems in *Drosophila* and *Saccharomyces*, while few species in other eukaryotic groups have been investigated^2^. Unlike in plants or animals, the sexual spores produced in many fungi can be directly cultured and examined for their viability, making fungi ideal organisms to study post-zygotic reproductive isolation. In addition, laboratory studies with fungal spores can permit the inclusion of large sample sizes and multiple experimental repeats, making inferences about the impacts of various environmental factors on spore germination feasible. However, very little is known about the factors that influence sexual spore viability and post-zygotic reproductive isolation in fungi^3^.

The *Cryptococcus neoformans* species complex (CNSC) has become a model for understanding fungal pathogenesis and fungal genetics. Members of this species complex are the major pathogens responsible for fungal meningitis, especially among immunocompromised individuals. The CNSC consists of two divergent species, *C. neoformans* and *C. deneoformans*, and their associative hybrids. Of the two species, *C. neoformans* dominates clinical populations, and has a global distribution – but is particularly prevalent in sub-Saharan Africa and Asia^4^,^5^,^6^. In contrast, *C. deneoformans* is more commonly isolated in Europe and is generally less virulent in animal models than *C. neoformans*^7^,^8^,^9^. It is estimated that *C. neoformans* and *C. deneoformans* have diverged from each other for over 18 million years^10^.

Despite the long-term divergence, there is little evidence of pre-zygotic reproductive isolation between *C. neoformans* and *C. deneoformans*. Many strains of these two species can mate relatively easily under laboratory conditions and hybrids are commonly found in both natural environments and in clinical samples^11^,^12^,^13^. Interestingly, most hybrids from natural populations or laboratory crosses are aneuploid or diploid, and are heterozygous at multiple loci, while the parental strains are haploid^14^,^15^,^16^. This is consistent with chromosome nondisjunction during meiosis^17^,^18^. The fact that hybrid offspring often display traits that differ significantly from both parent populations is of practical concerns for human pathogens. In the case of hybrid vigor, select individuals with these traits can be advantageous within certain ecological niches^19^. For example, certain hybrids have been found to be tolerant/resistant to high levels of anti-fungal drugs^19^,^20^, UV radiation^12^, and high temperatures^21^. Such fitness advantages have likely contributed to the broad distribution of CNSC hybrids in both their geographical ranges and ecological niches.

Basidiospores from hybrid crosses have been shown to have low viability, with only 5–20% of basidiospores germinating into mature colonies under standard laboratory conditions^14^,^18^. These results suggest that there is significant post-zygotic reproductive isolation between *C. neoformans* and *C. deneoformans*. However, at present, only a small proportion of non-viable basidiospores from between *C. neoformans* and *C. deneoformans* crosses could be explained by the classical Bateson-Dobzhansky-Muller (BDM) interactions^3^. Thus, other genetic factors or environmental factors must play a role in the low basidiospore germination rate in hybrid crosses. Gock *et al*. showed that the germination rates of various Ascomycete spores (e.g. *Aspergillus penicillioides*, *Penicillium roqueforti*) were influenced by environmental factors such as temperature and water activity^22^. Similarly, extracts from the substrate of the button mushroom, *Agaricus bisporus*, have been found to facilitate basidiospore germination^23^. The potential effects of environmental factors on basidiospore germination in CNSC have yet to be examined. Given that environmental factors can impact a diversity of physiological and life history traits in fungi, including CNSC^22^,^23^,^24^, we hypothesize that environmental factors will impact basidiospore germination in CNSC and that different environmental factors may influence intra-specific crosses differently than inter-specific crosses.

The objectives of this study were to examine the extent of basidiospore germination rate differences within and between the two species of the CNSC. Specifically, we selected five strains with known genome structure differences to construct six different crosses, including three intra-specific crosses and three inter-specific crosses. Basidiospores from these crosses were plated onto two different media and incubated at three temperatures to examine the potential influences of temperature and medium on basidiospore germination rates.

## Results

**Figure 1:**
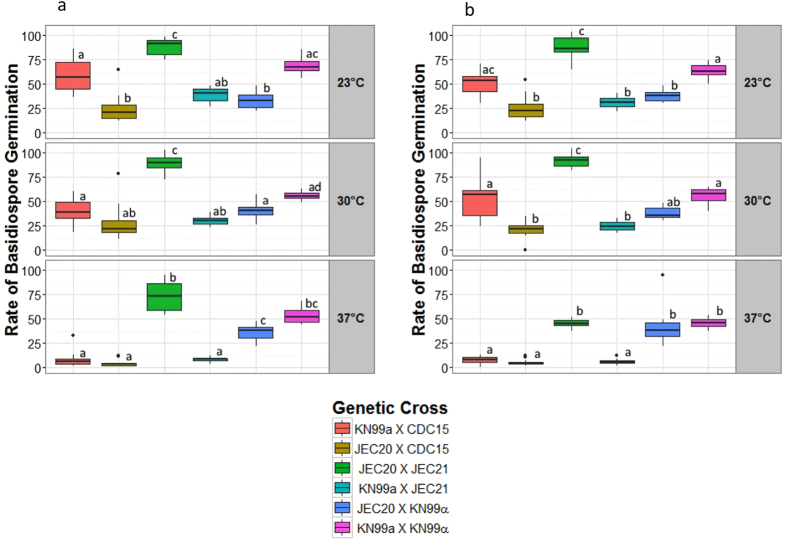
The distribution of germination rates of *Cryptococcus* basidiospores on MM (**a**) and YEPD (**b**) agar media, separated by incubation temperature shown at right of pane. Tukey groups, as denoted by the characters above each box, demonstrate significant differences between measures of germination rate. Offspring of crosses between isogenic parents generally had greater germination rates than those of non-isogenic parents – especially at higher temperatures.

In this study, we examined the rates of basidiospore germination from six crosses between strains in the human fungal pathogen *C. neoformans* species complex. The basidiospores were plated on two different media (a nutrient-rich YEPD medium and a nutrient poor minimal medium) and incubated at three different temperatures (23 °C, 30 °C and 37 °C). For most of the crosses and treatments, only one method was used to obtain basidiospores to estimate basidiospore germination rates. However, for two of the crosses, an additional method using a micromanipulator was used to isolate basidiospores, though these basidiospores were only incubated at one temperature and on one medium. A summary of basidiospore germination rates is presented in Table 1 and statistical significance of their differences is shown in Fig. 1. Below we describe the influences of the examined genetic and environmental factors on basidiospore germination among our crosses.

### Comparison of Spore Dissecting Methods

In this study, the sexual spores of CNSC were isolated by either directly picking basidiospores using a micromanipulator and placing them on fresh agar plates, or through gently washing sexual hyphae/basidiospores with a surfactant Tween 20 solution and then spread them on fresh agar plates. Basidiospore germination rates in two crosses (JEC20a X KN99α and KN99a X JEC21α) were assessed using both methods (Table 1). Using the micromanipulator, a total of 547 and 415 basidiospores were dissected from crosses JEC20a X KN99α and KN99a X JEC21α, respectively. The dissected basidiospores were placed on the rich YEPD medium and incubated at 23 °C. In one cross, JEC20a X KN99α, the germination rate of micro-dissected basidiospores (17.0%) was significantly lower than that obtained using the spread-plating method (38.48%; p < 0.005). However, there was no significant difference between the two spore isolation methods for basidiospore germination rates from the other cross, KN99a X JEC21α (29.16% vs. 31.7%; p > 0.05). Interestingly, as shown in Table 2, there was a wide variation in germination rates among basidiospores from different basidia within each of the two crosses.

### Effects of Temperature

Our analyses showed that temperature had a notable influence on basidiospore germination and that the effects differed among the crosses (Tables 1 and 3). The highest rate of germination was found for basidiospores from the intra-species cross, JEC20a X JEC21α at 23 °C and 30 °C, followed by that of another intra-specific cross KN99a X KN99α at 23 °C. Both of the above two crosses involved isogenic strain pairs. Interestingly, in the other intra-specific cross, KN99a X CDC15α, while basidiospore germination rates at 23 °C were comparable to that of KN99a X KN99α, there was a significant reduction in germination rates at 37 °C. Specifically, less than 10% of the basidiospores germinated at 37 °C, over a five-fold reduction compared to rates at the other temperatures.

Of the three inter-specific crosses, one (JEC20a X KN99α) showed relatively little change among the three temperature treatments while the other two crosses (JEC20a X CDC15α and KN99a X JEC21α) showed significant reduction in germination at 37 °C in comparison to rates at 23 °C and 30 °C. The lowest germination rate (~5%) was recorded for progeny from the inter-specific cross JEC20a X CDC15α under 37 °C.

Overall, our statistical analyses showed that temperature had significant effects on basidiospore germination in five of the six crosses (Table 3). The values of η^2^ in Table 3 show the amount of variance in basidiospore germination rate that could be attributed to changes in temperature. In three of the six crosses (KN99a X CDC15α, JEC20a X JEC21α, KN99a X JEC21α), temperature explained more than 50% (67%, 55.6%, 81.7%, respectively) of the observed variance in basidiospore germination rates. For two of the three remaining crosses, JEC20a X CDC15α and KN99a X KN99α, temperature showed smaller but still significant effects, contributing to 40.9% and 46.7% of the total variance respectively. The only cross that temperature did not show a significant effect on basidiospore germination was JEC20a X KN99α, for which η^2^ was 2.5%.

**Figure 2:**
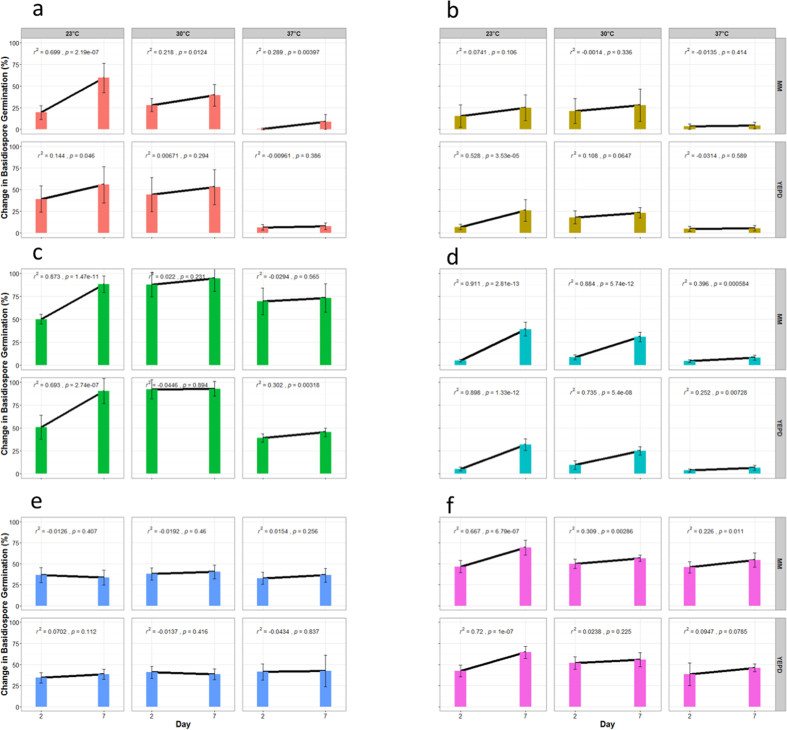
The changes in basidiospore germination rate from day 2 to day 7 following inoculation for all crosses. (**a**) KN99a X CDC15; (**b**) JEC20 X CDC15; (**c**) JEC20 X JEC21; (**d**) JEC20 X KN99α; (**e**) KN99a X JEC21; (**f**) KN99a X KN99α. The experimental conditions are labeled at the top and the right of the panes. Statistical significance of the difference in germination rates between days 2 and 7 in each of the panes is included.

The above comparisons were based on total basidiospore germination over the seven-day period. Interestingly, in addition to influencing the proportion at which basidiospores germinate at day seven, temperature was also found to affect the timing of spore germination (Fig. 2; Table 3). At 37 °C, most germinated basidiospores formed visible colonies within two days of incubation. However, in several of the crosses, a significant number of basidiospores germinated only after more than two days of incubation (Fig. 2). For example, at 23 °C, a relatively small number of basidiospores germinated in the inter-species cross JEC20a X KN99α within two days of incubation. In contrast, in JEC21α X KN99a, a cross with parental strains having the same genome structures as those of JEC20a X KN99α except at the mating types that were from alternative species, we found that most germinated basidiospores formed visible colonies within two days of incubation under the same conditions (Fig. 2; Table 3). Taken together, these results suggest that temperature had a cross-specific temporal effect on basidiospore germination.

### Effects of Medium on Basidiospore Germination

In contrast to the large effects that temperature has on basidiospore germination rates, relatively minor differences were observed between these two media. Specifically, there was no difference between YEPD and MM on basidiospore germination rates at each of the three temperatures for three (KN99a X CDC15α, JEC20a X CDC15α, and JEC20a X KN99α) of the six crosses. For the remaining three crosses (JEC20a X JEC21α, KN99a X KN99α, KN99a X JEC21α), while a statistically significant contribution of media to basidiospore germination rate differences was observed, media only accounted for about 5% or less of the total observed variance (Table 3). Interestingly, in all three of these crosses, germination rates were generally higher on the minimal medium than on the rich YEPD medium (Tables 1 and 3). The largest difference contributed by the media treatment was found for the isogenic cross of JEC20a X JEC21α at 37 °C for which, basidiospore germination rate was 73% on MM and only 45% on YEPD (p = 0.00373). In addition, there was a significant temperature–media interaction effect on basidiospore germination rate among progenies from this cross (p = 0.0005). However, such an interaction effect was not observed in other crosses.

Different from the notable effects of temperature on the temporal patterns of basidiospore germination among some of the crosses, the effect of medium is again relatively minor. Progeny from most crosses showed a similar pattern of basidiospore germination between days two and seven on the two media (Fig. 2). The only significant difference was observed for cross KN99a X CDC15α where progeny showed a significantly delayed germination on MM as compared to the rich YEPD medium (Fig. 2).

### Effects of Genome Structural Differences

The chromosomal structural differences between pairs of strains were calculated based on information presented in Sun and Xu^17^. In our analyses of the effect of genome structural differences on germination rate, the MAT loci were excluded from syntenic ratio calculations as these sex-determining regions are necessary for mating to occur between these strains. Thus, both isogenic crosses JEC20a X JEC21α and KN99a X KN99α were considered to have syntenic ratios of 1. The third intra-specific cross KN99a X CDC15α had a syntenic ratio ~0.94, with the notable difference between these two strains coming from the translocation between Chromosomes 3 and 11^17^. The hybrid crosses, KN99a X JEC21α and JEC20a X KN99α, differed at every genomic rearrangement shown in Sun and Xu^17^ and they have a syntenic ratio of ~ 0.76. Finally, the last strain pair CDC15α and JEC20a has a syntenic ratio of 0.82.

**Figure 3:**
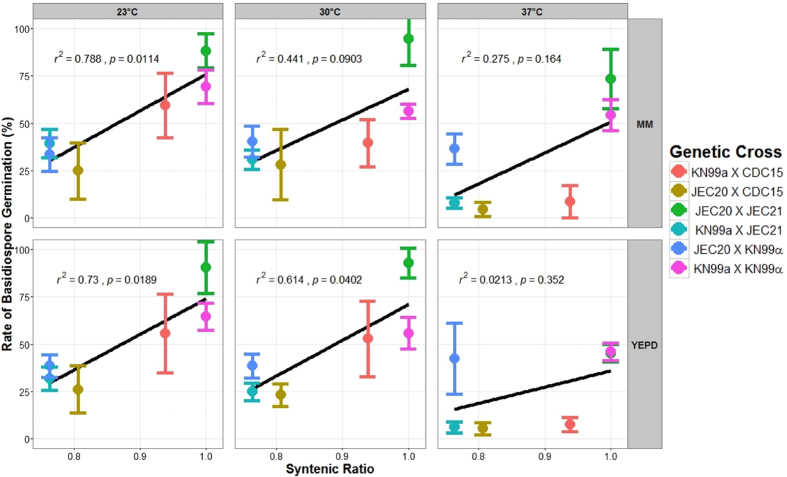
The relationship between syntenic ratio between parental strains and basidiospore germination rate among the six crosses at each of the six conditions. The experimental conditions are labeled at the top and the right of the panes.

Because of the significant influences of temperature and medium on basidiospore germination rates in *C. neoformans*, we used a linear model analysis for each of the six temperature-media combinations to determine the relationship between basidiospore germination rates and the syntenic ratios of parent strains among the crosses (Fig. 3). Under 23 °C, basidiospore germination rate was strongly correlated with syntenic ratio (r^2^ = 0.788 and 0.73 on MM and YEPD media respectively; p < 0.05). However, the strength of this correlation was reduced at higher temperatures, with r^2^ = 0.441 (on MM; p = 0.09) and 0.614 (on YEPD; p = 0.04) at 30 °C; and 0.275 (on MM; p = 0.164) and 0.0213 (on YEPD; p = 0.352) at 37 °C respectively. Taken together, our results suggest that the relationship between genome structure similarity and basidiospore germination was highly dependent on environmental conditions.

## Discussions

### Influence of Spore Isolation Methods

Prior to this study, there have been several reported estimates of basidiospore germination rate for the *Cryptococcus neoformans* species complex. These rates have shown to be highly variable, from about 5.5%^14^ and 19%^18^ to 69%^25^. The low rates (5.5–19%) were found in progeny from hybrid crosses between *C. neoformans* and *C. deneoformans* while the high rate was found in an intra-specific cross within *C. neoformans*. All these studies used microdissection to examine basidiospore germination. In this study, the manual dissection of basidiospore chains allowed for tracking and indexing of individual basidiospores in two crosses, providing fine germination estimates of basidiospore germination rates from individual basidia. Our results showed that basidiospores from different basidia of the same cross could have very different germination rates, from 0% to 100% (Table 2). Thus, large numbers of basidia and basidiospores need to be dissected in order to accurately estimate basidiospore germination rates. On the other hand, due to its ease of operation, spread-plating of a large number of basidiospores in spore suspensions can overcome the problem of large differences in germination rates among basidia within a cross. As a result, it could potentially provide an overall more robust estimate of basidiospore germination rate for each cross under each test condition for a broad set of conditions.

At present, the reason(s) for the difference between these two methods for cross JEC20a X KN99α is not known. One potential reason for the relatively higher rate of basidiospore germination for the spread-plated basidiospores was that these basidiospores were submerged in Tween 20 solutions before plating. A previous study showed that submerging basidiospores of the pine rust pathogen *Cronartium quercuum* f. sp. *fusiforme* in water before plating on agar significantly enhanced the spore germination rates, likely due to the release of inhibitory compounds from spore surfaces^26^. In addition, Tween 20 is a nonionic surfactant and mild detergent that can help solubilize basidiospore surface molecules and may enhance their germination. However, since the spore germination rates by the two methods for the other cross KN99a X JEC21α did not show a significant difference, our results could also be explained by the stochastic effects based on which basidia were picked using the micro-dissection method. As shown in Table 2, there was a big variation among basidia within each of the two crosses in the percentages of spores that were germinated. Below we mainly discuss the results obtained using the spread-plating method.

### Effects of Temperature and Medium

Tolerance to environmental stressors can be advantageous for organisms in natural environments. For human pathogens, tolerance to high temperatures is crucial for sustained infection of an endothermic host. In this study, we found that high temperature had a significant inhibitory effect on the germination of basidiospores from both intra-specific and inter-specific crosses. This result is different from that in the coprophilous fungus *Coprinus radiatus* where a significantly greater proportion of basidiospores germinated at the high temperature of 45 °C than at the low temperatures of 30–35 °C^27^. Interestingly, the inhibitory effect of 37 °C on basidiospores of CNSC was greater on progeny from inter-specific crosses (*e.g.* as shown in two of the three examined inter-specific crosses) than those from intra-specific crosses (*e.g.* as shown in one of the three intra-specific crosses) (Table 1).

At present, the reasons for the divergent rates of basidiospore germination among the different temperature conditions and among crosses are largely unknown. Using both site-directed and random mutagenesis, a recent study identified about 50 genes essential for basidiospore germination in *C. neoformans*^28^. The genes belong to diverse functional categories and include those involved in mitochondrial maintenance and function, cell division and cell cycle control, and the syntheses of membrane ergosterol and the cell wall. However, all those essential genes are found in the genomes of both *C. neoformans* and *C. deneoformans* and most of these genes are also present in other fungi^28^. Regardless, understanding their expression patterns and the detailed molecular and cellular processes that these genes exert in controlling basidiospore germination may help us reveal the divergent rates of basidiospore germination among the crosses and temperature conditions observed here. Since all five strains in our study are wild type and contain all the functional copies of these genes, our results suggest that other factors such as gene-gene interactions, strain-strain interactions, and/or genetic-environment interactions likely play important roles in determining the basidiospore germination rates in CNSC.

Most previous studies of basidiospore germination rates have examined only one cross each and thus the potential genotype-environment as well as strain-strain interaction effects between crosses could not be identified^14^,^18^,^24^,^25^,^26^,^27^,^28^. Interestingly, the large differences in spore germination rates at 37 °C between reciprocal inter-specific crosses (KN99a X JEC21α and JEC20a X KN99α) suggest that mating type combinations likely plays a role, with the combination of MATα from *C. neoformans* and MATa from *C. deneoformans* generating basidiospores that can germinate more easily at 37 °C than the parental combination of MATa from *C. neoformans* and MATα from *C. deneoformans.* This observation is also consistent with the predominance of *C. neoformans* MATα strains in clinics and human patients^13^.

Alternatively, the progeny mitochondrial type might also have played a role. Progeny mitochondrial DNA in inter-specific crosses in CNSC are predominantly inherited from the MATa parent^29^,^30^,^31^. Consistent with previous findings, our results indicated that progeny from the KN99a X JEC21α cross inherited the KN99a mitochondrial DNA while those from the JEC20a X KN99α inherited the JEC20a mitochondrial DNA (data not shown). Thus, it’s possible that the higher germination rate of progeny from the JEC20a X KN99α cross than the KN99a X JEC21α cross at 37 °C could have been due to the mitochondrial genotype from the JEC20 parent being able to better support basidiospore germination than the KN99a mitochondrial genotype. However, such a mitochondria-specific effect from the JEC20a parent was not observed for progeny from cross JEC20a X CDC15α where a low basidiospore germination rate was observed at 37 °C. Similarly, despite inheriting the KN99a mitochondrial genotype, progeny from cross KN99a X KN99α showed a high germination rate at 37 °C. Thus, if there were an effect of mitochondrial genotype on basidiospore germination, such as an effect were likely exerted through interacting with other genetic factors in the nuclear genome and seemed environment-specific.

Similar to the patterns of temperature effects on the overall rate of basidiospore germination, we observed little consistent effects of individual parental strains on the temporal pattern of basidiospore germination. Instead, most of the temporal variations could be attributed to strain-pair specific effects. Similarly, the reciprocal congenic crosses JEC20a X KN99α and KN99a X JEC21α showed large differences in their temporal patterns at all three temperatures, consistent with a role for the mating type locus combination in determining the timing of basidiospore germination under specific conditions.

Overall, our results showed that media could have a significant effect on basidiospore germination in certain crosses. Interestingly, in crosses where significant differences in basidiospore germination rates were found, the minimum medium tended to support a greater germination rate than the rich YEPD medium (Table 1). Our results suggest that limiting the supply of carbohydrates and amino acids can enhance the germination of basidiospores in the *C. neoformans* species complex. A slightly different but similar phenomenon has been reported for a variety of oligotrophic microorganisms, where limiting nutrient supplies can lead to enhanced microbial growth^32^,^33^. Given that the natural conditions that allow for hybridization within the CNSC likely have limited supply of free carbohydrates and free amino acids, our results are consistent with the hypothesis that the CNSC might have adopted a basidiospore germination strategy that required limited amount of organic compounds. Further experiments may lead to identify an optimal medium (media) that will support a greater rate of basidiospore germination than the two media we tested here.

Within the human host, the basidiospore germination condition likely differs from those on artificial media and in nature. In addition to the high temperature (at 37 °C) and an environment different from their natural ecological niche, the pathogen basidiospores have to face host defenses. Furthermore, these conditions can change as cryptococcal infection progresses from the respiratory tract to the bloodstream and the central nervous system^34^. Thus, the basidiospore germination rate inside hosts may be much lower than what we observed here on artificial media at 37 °C. The importance of environmental conditions on the fitness of CNSC was also demonstrated in an earlier study where transgressive hybrids showed greater fitness advantage at the high temperature and limiting nutrient environment (37 °C and MM) than at the low temperature and high nutrient environment (23 °C and YEPD)^19^. Together, these results call into question of the common assumption that basidiospores are the most important infectious propagules in CNSC. Instead, given the low germination rate of basidiospores at 37 °C, the roles of desiccated vegetative cells during infection could also be important.

### Influences of Genome Structure on Spore Germination

In general, we found that the more similar the parental strains are in their chromosomal structure, the greater the rate of their progeny basidiospore germination. This result is consistent with our expectation that genome synteny contributes to basidiospore viability. Similar results have been found in other organisms^35^,^36^. For example, in the *Saccharomyces cerevisiae* species complex, chromosomal structural differences contributed significantly to post-zygotic reproductive isolation among the closely related species^35^,^36^,^37^. Such contributions were at least partly due to chromosome non-disjunction during meiosis I that generated spores without certain essential chromosomal regions. Indeed, evidence for non-disjunction has been reported in hybrid crosses in CNSC^18^. In addition, evidence for multiple genetic incompatibilities influencing hybrid basidiospore viability between *C. neoformans* and *C. deneoformans* has also been reported^3^.

Among the six crosses, of special note is the intra-specific cross KN99a X CDC15α. The two parental strains in this cross have an estimated syntenic ratio of ~94% but with a reciprocal translocation between Chromosomes 3 and 11^17^. Interestingly, progeny from this cross had comparable germination rates as the isogenic cross KN99a X KN99α at 23 °C and 30 °C environments but were significantly lower at the 37 °C environment. The results suggest that the translocation might have a high-temperature specific effect on basidiospore germination in this cross. However, aside from the main reciprocal translocation, the translocated regions also contained several small-scale rearrangements between *C. neoformans* and *C. deneoformans*^17^ that could further contribute to low basidiospore viability at 37 °C.

As described above and shown previously^3^,^17^, neither chromosomal non-disjunction nor BDM genetic incompatibility (or a combination of both) are sufficient to explain the observed low basidiospore germination in several of our crosses and incubation conditions. In addition, the germination rates for most crosses were highly dependent on environmental conditions, with temperature being a major factor in our analyses. These results suggest that basidiospore germination and post-zygotic reproductive isolation in the CNSC is highly dependent on environmental conditions. Environmental-specific, parental-genotype influences of basidiospore germination are of particular interest for research on genetic incompatibilities that arise from genomic rearrangements, as genes that flank breakpoints can have modified expression levels^38^. Indeed, gene expression and repression under high temperature treatments have previously been described within CNSC^39^,^40^ and have been demonstrated to be crucial for sustained growth *in vivo*^41^.

The expression of target genes such as those involved in basidiospore germination within a stressful environment (*e.g*. high temperature) may be impacted by the specific genetic incompatibilities that arise under these conditions. In our analyses, the correlation between syntenic ratio and germination rate was found to progressively decay as incubation temperature increased (Fig. 2). In particular, cross KN99a X CDC15α had basidiospore germination rates similar to those of the KN99a X KN99α cross at 23 °C and 30 °C, consistent with the expectation based on their high syntenic ratios. However, in both crosses involving strain CDC15α, there was a large decrease in basidiospore germination at 37 °C, suggesting that certain genetic feature(s) within CDC15α likely makes its sexual progeny particularly susceptible to low germination at high temperature. It should be noted that this phenomenon was not unique to strain CDC15α as progeny from cross KN99a X JEC21α also showed very low germination rate at 37 °C.

In contrast to the observation for progeny from the intra-specific cross KN99a X CDC15α, we found that progeny from an inter-specific cross (JEC20a X KN99α) showed a high germination rate at 37 °C (Table 1). The high germination rate was especially notable when compared to results from a comparable cross KN99a X JEC21α (except at the mating type locus) where relatively few basidiospores germinated at 37 °C. In CNSC, the mating type loci contain genes that are crucial for sexual reproduction and pathogenicity^42^. These regions differ significantly in structure and gene arrangements between opposite mating types of the same species but the differences were greater between members of the two different species^43^. Previous investigation of the inter-specific cross, JEC20a X CDC15α, did not identify BDM incompatibilities within the mating type regions^3^. However, along with the results from the cross KN99a X KN99α, the results from the above three crosses suggest that the MATα allele in KN99α might enable progeny to germinate more efficiently at high temperatures, either alone or in combination and interaction with other genes in the genome. Significant influences of genetic interactions between loci on post-zygotic reproductive isolation have been reported in fruit flies^44^. Further genetic analyses of the germinated basidiospores at 37 °C between the two reciprocal hybrid crosses (JEC20a X KN99α and KN99a X JEC21α) in our study are needed in order to identify the genes and their interactions in influencing basidiospore germination.

Aside from the contributions of genome structure differences, nucleotide sequence divergence between parental strains could also play a significant role to basidiospore germination rate differences between the intraspecific and interspecific crosses. Specifically, *C. neoformans* and *C. deneoformans* exhibit ~10% sequence divergence at the nucleotide level^17^. When homologous chromosomes with such divergent sequences pair with each other and cross over during meiosis, the highly conserved mismatch repair system will be frequently triggered, interfere with normal chromosome disjunction, and lead to faulty chromosome segregation and lethal chromosomal rearrangements/deletions^45^,^46^,^47^. Hybrid crosses between *Saccharomyces cerevisiae* and *Saccharomyces paradoxus* have shown to result in spontaneous DNA lesions and chromosomal rearrangements, resulting in vastly decreased spore viability compared to intraspecific crosses^48^. Furthermore, chromosomal rearrangements and nucleotide sequence divergence can act synergistically to increase the rate of non-disjunction and reduce spore viability in *S. cerevisiae* and *S. paradoxus*^45^,^48^,^49^. Whether a similar mechanism exists in CNSC awaits further investigation.

### Conclusions and Perspectives

This study described the patterns of basidiospore germination among six crosses within and between the two closely related species *C. neoformans* and *C. deneoformans.* In addition to examining the potential effects of known genetic differences between pairs of strains on offspring basidiospore germination, we also examined the effects of three temperatures and two media on the rates of basidiospore germination. Our analyses revealed that all examined factors (individual parental strain, strain pair, temperature, and medium) could impact basidiospore germination. As expected, progeny from inter-specific crosses generally have a lower germination rate than those from intra-specific crosses. However, environmental factors can significantly impact the pattern. Importantly, there were notable interaction effects between the examined factors, with the 37 °C causing a large deduction of basidiospore germination for two of the three inter-specific crosses.

Previous studies have suggested that basidiospores are the most likely infectious propagules in the pathogenic *Cryptococcus* species complex. Our results here suggest that the 37 °C environment were not very conducive for basidiospore germination in the majority of the crosses. In addition, the low basidiospore germination rate at 37 °C forms a stark contrast to the vigorous vegetative growths of all parental strains as well as their germinated progeny at this temperature^19^,^24^,^40^,^41^. Taken together, these results indicate that basidiospores might not be the most important infectious propagule as commonly assumed. Rather, airborne, desiccated vegetative cells may also play an important role in initiating host infection.

Aside from the implications on pathogenesis, our results also have implications on the evolution and speciation research in these and other fungi. Evidence for both recent and potentially ancient hybridizations between *C. neoformans* and *C. deneoformans* have been reported^10^,^16^,^50^. In laboratory settings, there has been little evidence for pre-zygotic reproductive isolation between *C. neoformans* and *C. deneoformans* as mating can be easily induced between many strains of these two species. Instead, post-zygotic reproductive isolation is common, as shown here and in previous studies. However, the BDM genetic incompatibilities identified based on one basidiospore-germination condition (23 °C on YEPD) likely explains only a subset of the incompatibilities between these two species. Additional incompatibilities that are unique to a specific environmental condition must also exist. For example, genic interactions between specific loci near translocated regions could play a significant role in offspring inviability during high temperature growth^51^. The locations of those genetic factors and how they interact with each other await further investigation.

## Materials and Methods

### Strains

Four laboratory strains and one clinical isolate were used in this study. The four laboratory strains correspond to two pairs of isogenic isolates, with one pair, JEC20a and JEC21α, belonging to *C. deneoformans* (serotype D); and another pair, KN99a and KN99α, belonging to *C. neoformans* (serotype A). The isogenic strain pairs differ only at the mating type locus^52^,^53^,^54^. Strains JEC20a and KN99a belong to mating type a while strains JEC21α and KN99α have the α mating type. The clinical isolate used in this study was CDC15α, of *C. neoformans* (serotype A)^15^,^17^. CDC15α was obtained in a national survey by the US Center for Disease Control and Prevention^55^ and is known to differ from KN99a and KN99α strains by large scale genomic rearrangements involving Chromosomes 3 and 11^17^.

### Crosses

The two MATa and three MATα strains were used to create six crosses. Three of the crosses were between strains within the same species: one intra-specific cross was within *C. deneoformans* (JEC20a X JEC21α) and two were within *C. neoformans* (KN99a X KN99α, KN99a X CDC15α). The remaining three crosses (JEC20a X CDC15α, KN99a X JEC21α, JEC20a X KN99α) were inter-species, between strains of *C. deneoformans* and *C. neoformans*.

In preparation for mating, cells stored at −80 °C were first cultured on yeast extract peptone dextrose (YEPD) agar medium and incubated at 30 °C for five days. Actively growing cultures were then re-suspended in sterile distilled water and adjusted to a concentration of 10^5^ cells/μl. For each cross, 50 μl of the adjusted cell suspension from each of the two parents was thoroughly mixed together. The mixed cell solutions were spotted onto separate plates containing V8-juice agar, a specific media to induce sexual mating in CNSC^56^. Each plate contained three spots of the mixed parental cells and one spot for each of pure parental cells as negative controls. Each spot contained 10 μl of the cell suspension, equivalent to about 10^6^ cells. In total, 30 mating plates were prepared and incubated at 23 °C for four weeks to allow for mating and sexual spore formation. For species within the CNSC, a successful mating is indicated by the formation of hyphae along the periphery of the parental yeast colony. The hyphae typically extend away from the original parental yeast cell spot, with the ends of these hyphae differentiating into basidia, the sexual structures of the CNSC, which subsequently produce chains of basidiospores^56^.

### Germination of Basidiospores

To determine basidiospore germination rates, two approaches were taken. The first approach examined basidiospores individually isolated using a micromanipulator (Singer Instruments, England). In this approach, the hyphae containing basidiospores were first identified using a microscope. The agar medium containing the hyphae was then cut using a sterile scalpel and transferred to a complementary space in a new plate containing YEPD medium. Multiple basidia from the section of transplanted agar, with the chains of basidiospores attached, were then picked and placed onto fresh areas of the plate. Basidiospores were then individually picked and transferred to pre-determined spots on the same agar plate. Plates containing dissected basidiospores were incubated at 23 °C for one week to ensure that any slow-germinating or slow-growing basidiospores could establish a visible colony. Because of the high workload involved with micromanipulation, only two crosses (crosses JEC20a X KN99α and KN99a X JEC21α) were examined by this method and the dissected basidiospores from this cross were only incubated on the rich medium YEPD at 23 °C. The spore germination rate was calculated as the number of visible colonies (*i.e*. germinated spores) formed divided by the total number of dissected basidiospores for each cross.

A second approach was used to examine the basidiospore germination rates of all six crosses at all six incubation conditions. In this approach, after four weeks of mating on V8-juice agar medium at 23 °C, sections of agar containing only hyphae and basidiospores (i.e. no parental yeast cells) were cut and transferred to a new blank plate and the hyphae and basidiospores were washed following the method outlined by Choi *et al*.^57^. Specifically, using a pipette, 50–100 μm of a sterile 0.5% Tween 20 solution (Sigma Aldrich: Mississauga) was applied to the mycelial surface of each agar block and spores were gently taken up along with the solution by the pipette and transferred to a sterile 1.5 ml micro-centrifuge tube. The spore solutions were examined using a light microscope to determine the density of basidiospores as well as to ensure the absence of hyphae in each solution; any spore suspensions containing hyphae were discarded. Basidiospore suspensions were then concentrated/diluted with additional 0.5% Tween-20 solution to a final density of approximately 2–3 × 10^3^ spores/ml. Diluted spore suspension were spread-plated on either the YEPD agar medium or the Minimal Medium (MM, yeast nitrogen base with ammonium sulfate but without amino acids). On each plate, 100 μl of basidiospore suspension was spread evenly over the agar surface using 1 mm diameter sterile glass beads.

Using the second approach, basidiospores from each of the six crosses were plated on a total of 72 plates with 36 containing the YEPD medium and 36 containing the MM media. Of the 36 plates, 12 were incubated at each of three temperatures (23, 30, or 37 °C). A total of 432 plates were used for the six crosses. The number of visible colonies formed by germinated spores was counted on each plate at two and seven days after incubation. All visible colonies were counted. The germination rate of basidiospores was determined as a ratio of the number of colonies observed to the estimated total number of basidiospores plated.

### Genome Structural Differences

Data on chromosomal structural differences among strains JEC21α, H99α, and CDC15α were obtained from Sun and Xu^17^. Since JEC20a is isogenic with JEC21α and both KN99a and KN99α are isogenic with H99α^53^,^54^, we assume that JEC20a and JEC21α have the same genome structure (except at the mating type locus) and that KN99a, KN99α, and H99α would have the same genome structure. Based on the chromosomal structural differences, we estimated the total percentage of non-syntenic blocks (including all known simple inversions, complex rearrangements, and translocations) over the whole genome between each of the six pairs of strains^17^.

### Statistical Analyses

Statistical significance of the basidiospore germination rate differences between crosses and the effects of genetic and environmental factors contributing to the differences were analyzed using multifactorial ANOVA, post-hoc Tukey Honest Significant Difference (HSD) tests, Pearson’s correlation, and η^2^ using R (V3.1.3; packages: stats, lsr, agricolae, ggplot2, cowplot)^58^.

## Additional Information

**How to cite this article**: Forsythe, A. *et al*. Genetic and environmental influences on the germination of basidiospores in the *Cryptococcus neoformans* species complex. *Sci. Rep.* **6**, 33828; doi: 10.1038/srep33828 (2016).

## Figures and Tables

**Table 1:** Mean rate of basidiospore germination after day seven for six genetic crosses of CNSC strains at six environmental conditions.

Genetic Cross	Temperature	Medium	Germination Rate (%)
KN99a X CDC15α	23 °C	MM	59.55 ± 16.98
		YEPD	55.51 ± 20.79
	30 °C	MM	39.60 ± 12.36
		YEPD	52.78 ± 19.93
	37 °C	MM	8.67 ± 8.57
		YEPD	7.74 ± 3.76
JEC20a X CDC15α	23 °C	MM	25.00 ± 14.82
		YEPD	26.15 ± 12.46
	30 °C	MM	28.21 ± 18.68
		YEPD	21.30 ± 8.98
	37 °C	MM	4.52 ± 3.76
		YEPD	5.47 ± 3.18
JEC20a X JEC21α	23 °C	MM	88.24 ± 9.07
		YEPD	90.55 ± 13.61
	30 °C	MM	94.66 ± 13.90
		YEPD	92.83 ± 7.94
	37 °C	MM	73.39 ± 15.60
		YEPD	45.19 ± 4.66
KN99a X JEC21α	23 °C	MM	39.24 ± 7.54
		YEPD	31.70 ± 6.17
			29.16^†^
	30 °C	MM	30.80 ± 5.14
		YEPD	24.85 ± 4.65
	37 °C	MM	8.12 ± 2.76
		YEPD	6.08 ± 2.80
JEC20a X KN99α	23 °C	MM	33.50 ± 8.85
		YEPD	38.49 ± 6.04
			17.00^‡^
	30 °C	MM	40.36 ± 8.18
		YEPD	38.49 ± 6.29
	37 °C	MM	36.46 ± 8.14
		YEPD	42.47 ± 18.68
KN99a X KN99α	23 °C	MM	69.26 ± 8.85
		YEPD	64.50 ± 7.23
	30 °C	MM	56.43 ± 3.79
		YEPD	55.76 ± 8.42
	37 °C	MM	54.36 ± 8.26
		YEPD	46.09 ± 4.61

**Table 2:** Detailed germination rate for spores from individually dissected basidia in two crosses.

Basidia Number	JEC20a X KN99α		KN99a X JEC21α	
	Nt	Ng (%)	Nt	Ng (%)
1	29	7 (24.1%)	16	0
2	1	0	1	0
3	8	8 (100%)	11	1 (9.1%)
4	23	3 (13.0%)	29	0
5	4	0	7	0
6	26	0	3	0
7	37	0	19	0
8	3	1 (33.3%)	27	0
9	26	6 (23.1%)	2	0
10	19	4 (21.1%)	22	0
11	5	2 (40%)	34	24 (70.6%)
12	15	3 (20%)	24	0
13	2	0	12	6 (50%)
14	23	8 (34.8%)	31	21 (67.7%)
15	30	13 (43.3%)	32	16 (50%)
16	16	0	8	7 (87.5%)
17	24	0	3	0
18	24	1 (4.2%)	24	0
19	19	15 (78.9%)	20	10 (50%)
20	42	9 (21.4%)	22	11 (50%)
21	26	0	39	24 (61.5%)
22	3	0	11	1 (9.1%)
23	19	0	18	0
24	23	0	—	—
25	29	0	—	—
26	47	13 (27.7%)	—	—
27	24	0	—	—
Total	547	93 (17.00%)	415	121 (29.16%)

Nt: the total number of dissected spores from the specific basidia; Ng: the number of spores germinated from the specific basidia.

**Table 3:** The results of a multiple ANOVA demonstrate the individual and combined effects of experimental factors on basidiospore germination.

Genetic Cross	Source of Variance	Degrees of Freedom	Germination Rate		Change in Germination Rate	
			p–value	η^2^	p – value	η^2^
KN99a X CDC15α	Temperature	2	***	0.67	***	0.396
	Media	1	0.427	0.003	***	0.053
	Temperature:Media	2	0.115	0.021	0.281	0.01
	Residuals	66	—	0.306	—	0.543
JEC20a X CDC15α	Temperature	2	***	0.409	***	0.252
	Media	1	0.565	0.003	0.132	0.012
	Temperature:Media	2	0.405	0.016	0.271	0.014
	Residuals	66	—	0.572	—	0.722
JEC20a X JEC21α	Temperature	2	***	0.556	***	0.147
	Media	1	**	0.050	0.171	0.011
	Temperature:Media	2	***	0.108	0.059	0.034
	Residuals	66	—	0.285	—	0.808
KN99a X JEC21α	Temperature	2	***	0.817	***	0.445
	Media	1	***	0.038	0.066	0.013
	Temperature:Media	2	0.170	0.008	0.871	0.001
	Residuals	66	—	0.138	—	0.541
JEC20a X KN99α	Temperature	2	0.413	0.025	0.716	0.005
	Media	1	0.214	0.022	0.432	0.004
	Temperature:Media	2	0.361	0.029	0.647	0.006
	Residuals	66	—	0.924	—	0.985
KN99a X KN99α	Temperature	2	***	0.467	0.284	0.018
	Media	1	*	0.051	0.397	0.005
	Temperature:Media	2	0.190	0.024	0.697	0.005
	Residuals	66	—	0.458	—	0.972

As a correction for large sample sizes, η^2^ has been included as a measure of effect size (N = 360).
